# Use of Ceftolozane/Tazobactam in a Case of Septic Shock by Puerperal Sepsis

**DOI:** 10.1155/2019/8463693

**Published:** 2019-05-29

**Authors:** Mario Pezzi, Anna Maria Scozzafava, Anna Maria Giglio, Renata Vozzo, Patrizia Dina Casella, Simona Paola Tiburzi, Lucio Cosco, Mario Verre

**Affiliations:** ^1^Intensive Care Department, General Hospital “Pugliese-Ciaccio”, Catanzaro, Italy; ^2^Infectious Diseases Department, General Hospital “Pugliese-Ciaccio”, Catanzaro, Italy

## Abstract

Multiresistant bacteria infections cause widespread morbidity and mortality and lead to an increase in expenses for hospital stays and complications. We describe the case of a 27-year-old patient with puerperal sepsis after cesarean section due to Escherichia coli complicated by multiresistant Klebsiella ESBL-producing superinfection with septic shock and multiple organ dysfunction syndrome, successfully treated with ceftolozane/tazobactam.

## 1. Introduction

The pregnant woman is exposed to sepsis. Indeed in this population, the incidence of bacteremia is high: 7.5/1000 of which 8 to 10% develop sepsis [1]. Bacterial contamination can occur for the transport of germs already present on the external genitalia of the woman. Content uterine contamination by vaginal flora is responsible for the majority of chorioamnionitis, endometritis, and parametritis. This type of ascending infection is favored by premature and/or prolonged membranes rupture, by vaginal infection, by maneuvering during delivery, or by cesarean section.

Propagation occurs through the endoluminal way, through the tubes towards the peritoneum, or more frequently, by venous route or lymphatic, reaching the laterouterine venous plexus, the lymphatics vessels of the broad ligament, the peritoneum pelvic.

The obstetric sepsis, in the majority of cases, develops secondarily to genital tract infections, in which the etiologic agents most common are Escherichia coli, Klebsiella pneumoniae, Enterobacter sp, enterococcus bacteria faecalis and anaerobics [2]. Puerperal sepsis should be suspected in case of oral temperature ≥38° Celsius, lasting at least 2 days, within 10 days of delivery, excluding first 24 hours in which there may frequently be a slight increase of the spontaneously resolving temperature. A WHO technical working group defined puerperal sepsis as infection of the genital tract occurring at any time between the onset of rupture of membranes or labor and the 42nd day postpartum in which two or more of the following are present: pelvic pain, fever, abnormal vaginal discharge, abnormal smell/foul odour discharge or delay in uterine involution [3].

Maternal sepsis is a life-threatening condition defined as organ dysfunction resulting from infection during pregnancy, childbirth, postabortion, or postpartum period [4]. Endometritis is the most common cause of postpartum infections with an incidence of 1-3% after vaginal birth and 5-15% after cesarean section with perioperative antibiotic prophylaxis [5]. The cesarean section is the most important risk factor for postpartum endometritis. Indeed, bacteremia complicates 14% of cesarean section performed for labor failure, especially in case of premature birth or in context of chorioamnionitis [6].

The incidence of potentially fatal septic shock in the peripartum varies between 1/8000 and 1/44000 [7].

In about 50% of patients with septic shock, the etiology is not identified of the infection; in patients with isolation from etiological agent, gram-negative bacilli 30-80% of the cases are identified, while gram-positive bacteria are isolated only from 5 to 25% of cases [8].

Sepsis still remains a leading cause of preventable maternal death worldwide [9]. The contribution of sepsis as a cause of maternal mortality is between 3 % in developed countries and 12 % in developing countries [10]. Puerperal sepsis and septic shock require immediate and appropriate early goal directed therapy to avoid maternal morbidity and mortality [11].

## 2. Clinical Case

**Figure 1:**
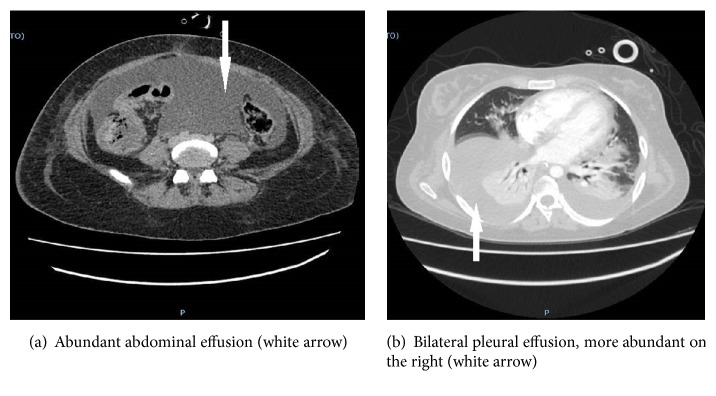


The 27-year-old patient at the fortieth week of gestation admitted to the obstetrics gynecology department underwent cesarean section in urgency due to premature rupture of membranes with heavy stained amniotic fluid and to the nonprogression of the fetus in the birth canal. A female infant weighing 3.2 kg was extracted from the uterus. The newborn presented an index of Apgar 10. After the extraction of the newborn and the uterus suture, repeated washings of the abdominal cavity were carried out. A sample of amniotic fluid was sent to the microbiology laboratory for a culture examination. The following day, the patient presented elevated fever (temperature trend is described in Figure 4), hypotension, and respiratory failure. For this reason she was transferred to intensive care unit (ICU). In the ICU, during the first 6 hours, the patient was subjected to fluid resuscitation with crystalloids to obtain the restoration of tissue perfusion and normalization of oxidative metabolism. Norepinephrine 0,1*μ*g/kg/min was administered to counteract hypotension. Respiration was supported with humidified High Flow Nasal Prong (HFNP). A therapy with broad spectrum antibiotics was established. The patient presented a marked Leucopenia: WBC: 0,740 cells/mm^3^ treated with filgrastim a granulocyte colony-stimulating factor (G-CSF) 10 mcg/kg once daily until the cells went back to 2,000/mm^3^ (the WBC count trend is described in Figure 3). The patient's blood culture test was positive for Escherichia coli. Also the culture tests on the baby's blood and on the amniotic fluid were positive for Escherichia coli. The newborn girl showed signs of sepsis and was given amikacin therapy 10 mg / kg IM loading dose followed with 7.5 mg / kg IM every 12 hours for seven days, resulting in complete recovery. Antibiotic therapy at the patient was set up with amikacin 15 mg/kg/day IV divided every 12 hours, levofloxacin 750 mg IV every 24 hours, and caspofungin 70 mg IV infusion on day 1, followed by 50 mg IV daily thereafter. Computed tomography showed a modest pleural effusion and an abundant abdominal effusion (Figure 1(a)).

In the following days the clinical conditions worsened. In the fourth day, endotracheal intubation and artificial ventilation were needed to progressively worsen respiratory dynamics with hypoxia. Computerized axial tomography showed bilateral pleural effusion, more abundant on the right, associated with bilateral parenchymal thickening (Figure 1(b)), abundant endoabdominal effusion, liver, kidneys, spleen, adrenal, and pancreas without obvious focal-type lesions in place overlaid by enteric material, the cecum and the ascendant, and by gas the transverse colon. Pleural drainage was inserted with extraction of 1100 ml of pleural fluid. The cultural exam was negative. In the sixth day the dehiscence of the surgical wound occurred, treated with hermetic dressing and negative pressure system (Vacuum Assisted Closure Therapy). In the seventh day from the culture exam on bronchoalveolar lavage, it was obtained positivity for acinetobacter baumannii sensitive to colimycin. In the same day there was a positivization of the urine culture and blood culture for Klebsiella Spp. Antibiotic therapy was therefore modified. Amikacin was substituted with colimycin IV 9 million units, divided into two doses. The presence of candida glabrata in the rectal pad was also found. Caspofungin was suspended and substituted with amphotericin B liposome 5 mg / kg IV daily.

The clinical situation improved. Infusion of norepinephrine was suspended gradually.

On the tenth day the patient completed the respiratory weaning and breathed spontaneously with high flows nasal prong. Right thoracic drainage was removed. After extubation a motor polyneuropathy was diagnosed, confirmed by the electromyographic examination. Therapy with immune globulin was started: 0.4 g / kg / day IV for 5 doses.

On the fifteenth day, high fever with hypotension and hypoxemia reappeared. The patient was again intubated and subjected to artificial ventilation. The infusion of norepinephrine was restored. It was detected a severe thrombocytopenia (7,000 cells/mm^3^) treated with platelet transfusions.

There was evidence of positive culture urine for multiresistant Escherichia coli and positive blood culture and the surgical wound for multiresistant Klebsiella species ESBL-producing (ESBL= extended-spectrum beta-lactamases).

**Figure 2:**
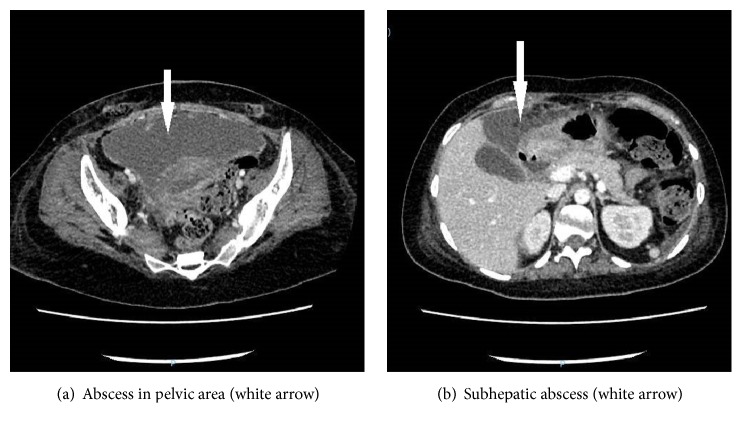


**Figure 3:**
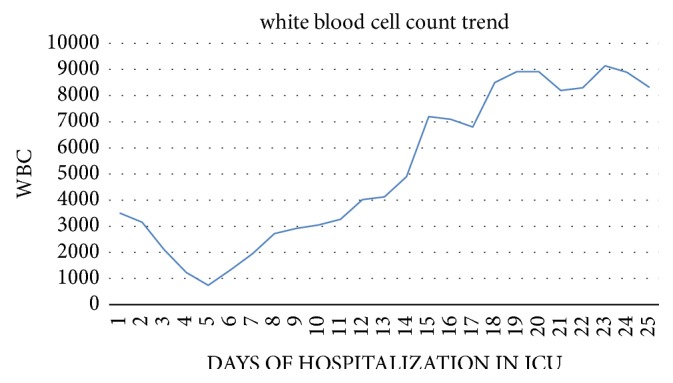
WBC count trend (the therapy with ceftolozane/tazobactam started on day 19').

**Figure 4:**
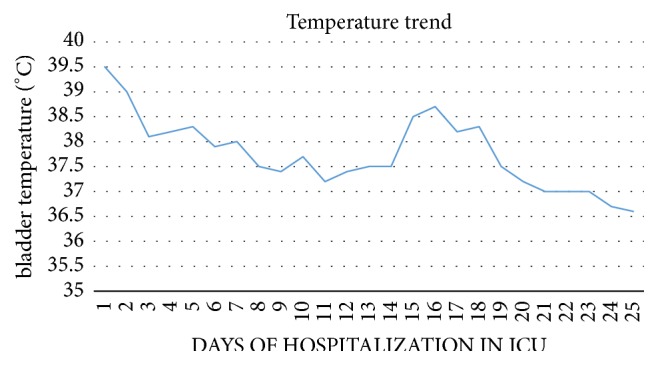
Temperature trend (the therapy with ceftolozane/tazobactam started on day 19').

The computerized tomography of the abdomen was repeated, which showed two abscesses, one in the pelvic area, before the bladder of 16x4 cm (Figure 2(a)) and another in the right subhepatic of 5x3 cm (Figure 2(b)).

An exploratory laparotomy was performed with evidence of diffuse purulent fibrinous peritonitis. Surgery was performed with complete cleansing of the abdominal cavity with abscess drainage and peritoneal lavage intraoperative.

Administration of levofloxacin was suspended and the administration of ceftolozane/tazobactam was started: 1.5 g (ceftolozane 1 g / tazobactam 0.5 g) IV infused over 1 hour every 8 hours for 14 days associated with metronidazole 500 mg IV every 8 hours.

In the following days the respiratory and hemodynamic parameters improved.

On the twenty-second day the patient completed the ventilatory weaning. The patient was alert, conscious in spontaneous breathing, and assisted by oxygen therapy with a facial mask. She no longer had a fever. The hemodynamic parameters were stable and diuresis preserved. Modest thrombocytopenia was found. Free feeding was by mouth with parenteral support for peripheral vein. The signs of polyneuropathy persisted.

Microbiological tests on urine, blood, and surgical wound became negative.

On the twenty-fifth day the patient was transferred to the gynecology hospital ward. The main events of the ICU stay are described in Table 1.

After another ten days of hospitalization, she was transferred to a rehabilitation center for the treatment of polyneuropathy, where she remained hospitalized for three months and from which she was discharged home after complete recovery.

## 3. Discussion and Conclusions

Multidrug resistant (MDR) bacteria are well-recognized to be one of the most important current public health problems. Typically, MDR bacteria are associated with nosocomial infections [12].

Infections caused by multiresistant microorganisms have a worse prognosis than the one caused from sensitive pathogens, since treatments with antimicrobial agents used empirically are not effective in a significant number of cases [13]. The increase in antimicrobial resistance, together with the poor development of new antibiotics (especially against gram negative), causes fewer therapeutic options for the treatment of these infectious diseases. In recent years, new antibiotic drugs have become available to fight multiresistant bacteria.

Ceftolozane/tazobactam is antibacterial combination drug and includes a cephalosporin and a beta-lactamase inhibitor. Ceftolozane is a cephalosporin whose base structure is similar to that of ceftazidime. The mechanism of action of ceftolozane is similar to that of other beta-lactam antibiotics; it exerts a bactericidal activity binding to penicillin binding proteins (PBP), which results in the inhibition of bacterial cell wall synthesis and subsequent cell death. Tazobactam is a sulfone, capable of binding irreversibly to the site of action of serine beta-lactamases [14].

Ceftolozane/tazobactam is indicated in adults for treatment of complicated intra-abdominal and urinary tract infections, including pyelonephritis, caused by susceptible gram-negative and gram-positive microorganisms: Enterobacter cloacae, Escherichia coli, Klebsiella oxytoca, Klebsiella pneumonia, proteus mirabilis, pseudomonas aeruginosa, Bacteroides fragilis, streptococcus anginosus, streptococcus constellatus, and streptococcus salivarius [15].

Complicated intra-abdominal infections (CIAIS) include a wide spectrum of pathological conditions, ranging from uncomplicated appendices at peritonitis, both localized (abscesses intra-abdominal) or diffuse. Contamination of the peritoneum can cause a perforation spontaneous (appendicitis, perforated ulcer or diverticulitis), surgery or trauma.

Effective treatment consists of a combination of early diagnosis, surgery appropriate and an empirical antibiotic therapy, broad spectrum.

The pathogens commonly responsible in the CIAIS are E. coli, enterobacteriaceae (e.g., proteus, Klebsiella spp), pseudomonas aeruginosae, and Bacteroides fragilis. The expression is increasing of ESBLs bacteria and resistant strains of P. Aeruginosae.

Ceftolozane/tazobactam in combination with metronidazole for 4 to 14 days in adults with complicated intra-abdominal infections was not inferior to meropenem for the clinical cure rate (complete resolution or significant improvement in signs and symptoms) 24 to 32 days following initiation of therapy (83% versus 87.3%) in 2 randomized trials (N=979). Infections included appendicitis, cholecystitis, diverticulitis, gastric, duodenal and intestinal perforation, abscesses, and peritonitis [16, 17].

The drug is rapidly bactericidal, inhibits cell wall synthesis, is active against organisms with porin deficiencies or mutations, inhibits *β*-lactamases, and broadens coverage to most extended-spectrum *β*-lactamases (ESBL) produced by enterobacteriaceae that hydrolyze a wide spectrum of beta-lactam drugs. Its potential benefit lies in its activity on certain resistant gram-negative bacteria, which can treat patients with suspected or documented infections with broad-spectrum beta-lactamase producing enterobacteria (ESBL) or pseudomonas aeruginosa, in the context of a carbapenem savings strategy. The drug is indicated as an alternative to carbapenems, in patients with risk factors for the presence of enterobacteria that produce ESBL, in order to avoid the development of Klebsiella producing carbapenemase highly drug-resistant [18].

Resistance to ceftolozane/tazobactam is conferred by the production of serine carbapenemases (Klebsiella pneumoniae carbapenemase or KPC) and metallo-beta lactamases. Although some beta-lactamase producing isolates of Escherichia coli and Klebsiella pneumoniae were susceptible to ceftolozane/tazobactam, other isolates were not susceptible at minimum inhibitory concentrations of greater than 2 mcg/mL, including enzyme groups: CTX-M (cefotaximase), OXA (oxacillinase), TEM (temoniera), and SHV-1(sulphydryl variable). Cross-resistance with other cephalosporins may occur. Recently evolved enzymes Klebsiella pneumoniae carbapenemase (KPC-2) are potentially resistant to tazobactam, which in fact loses much of its activity to MDR enterobacteria [14].

In these cases the use of other antibiotics as ceftazidime–avibactam is indicated [19].

New antibiotics or combinations of antibiotics associated with beta-lactamase inhibitors are undergoing clinical trials: cefiderocol, eravacycline, plazomicin, murepavadin, aztreonam/avibactam, meropenem/vaborbactam, and imipenem-relebactam [20].

In agreement with Italian Study Group on Resistant Infections of the Italian Anti-Infective Therapy Society, we believe that the drug has a special value for clinicians in suspected or documented severe infections due to MDR P. aeruginosa and an alternative to carbapenems for the treatment of infections caused by ESBL-producers, thus allowing a carbapenem-sparing strategy [21].

In the case described by us, sepsis was initially due to Escherichia coli and subsequently to ESBL-producing Klebsiella spp.

The use of ceftolozane / tazobactam, associated with metronidazole, alternative to carbapenems, has been shown to be effective in the treatment of life-threatening generalized puerperal sepsis with septic shock and multiple organ dysfunction syndrome.

## Figures and Tables

**Table 1:** Main clinical events during ICU hospitalization.

DAY OF HOSPITALIZATION IN ICU	MAIN CLINICAL EVENTS
1	ICU Admission; start infusion of norepinephrine
2	
3	Patient's blood culture test positive for Escherichia coli
4	Start of invasive mechanical ventilation; thoracic drainage
5	Severe neutropenia (WBC: 0,740 cells/mm^3^)
6	Dehiscence of the surgical wound
7	Bronchoalveolar lavage (positivity for Acinetobacter baumannii)
8	
9	Stop infusion of norepinephrine
10	Respiratory weaning and extubation
11	
12	Diagnosis of motor polyneuropathy
13	
14	
15	Respiratory failure; restart of invasive mechanical ventilation
16	Hypotension with restore of norepinephrine infusion
17	Severe thrombocytopenia treated with transfusion
18	Diagnosis of multiple abdominal abscesses
19	Start therapy with ceftolozane/tazobactam
20	Exploratory laparotomy with abscesses drainage
21	
22	Respiratory weaning with extubation; stop norepinephrine infusion
23	
24	
25	ICU Discharge
